# Microstructural Analysis of Thermally Treated Geopolymer Incorporated with Neodymium

**DOI:** 10.3390/nano13101663

**Published:** 2023-05-18

**Authors:** Sanja Knežević, Marija Ivanović, Dalibor Stanković, Danilo Kisić, Snežana Nenadović, Jelena Potočnik, Miloš Nenadović

**Affiliations:** 1 Department of Materials, Vinča Institute of Nuclear Sciences, National Institute of the Republic of Serbia, University of Belgrade, Mike Petrović Alasa 12-14, Vinča, 11000 Belgrade, Serbia; sanja.knezevic@vin.bg.ac.rs (S.K.); marija@vin.bg.ac.rs (M.I.); msneza@vin.bg.ac.rs (S.N.); 2 Faculty of Chemistry, University of Belgrade, Studentski trg 16, 11000 Belgrade, Serbia; dalibors@chem.bg.ac.rs; 3 Department of Atomics Physics, Vinča Institute of Nuclear Sciences, National Institute of the Republic of Serbia, Mike Petrović Alasa 12-14, Vinča, 11000 Belgrade, Serbia; dankisic@vin.bg.ac.rs (D.K.); jpotocnik@vin.bg.ac.rs (J.P.)

**Keywords:** geopolymer, Nd_2_O_3_, rare earth, DRIFT, SEM, TEM, XPS

## Abstract

The following investigation presents the thermal treatment of geopolymer at 300 °C, 600 °C and 900 °C. We investigated what happens to the geopolymer base when incorporated with 1% and 5% of neodymium in the form Nd_2_O_3_. A total of six samples were synthesized. Geopolymer 1 contained 1% and geopolymer 2 contained 5% Nd_2_O_3_, and these samples were treated at 300 °C; then, samples geopolymer 3 and geopolymer 4 also had the same percentage composition of Nd_2_O_3_ and were treated at 600 °C, while samples geopolymer 5 and geopolymer 6were treated at 900 °C. Physical and chemical changes in the aluminosilicate geopolymer matrix were monitored. The incorporation of rare earths into the polymer network of aluminosilicates has been proven to disrupt the basic structure of geopolymers; however, with increased temperatures, these materials show even more unusual properties. *Diffuse reflectance infrared Fourier transform* (DRIFT) analysis showed that the intensity of the vibrational band decreases with the increase in temperature during thermal treatment, suggesting alterations in the chemical structure of the geopolymers. *Transmission electron microscopy* (TEM) analysis showed that the diameter of the nanoparticles containing Al_2_O_3_ is in the range 5–10 nm, while larger crystallites range from 30 to 80 nm. *Scanning electron microscopy* (SEM) analysis revealed that the temperature of the thermal treatment increases to 300 °C and 600 °C; the porosity of geopolymer increases in the form of the appearance of large pores and cracks in material. *X-ray photoelectron spectroscopy* (XPS) analysis was used to investigate the surface chemistry of geopolymers, including the chemical composition of the surface, the oxidation state of the elements, and the presence of functional groups. The UV/Vis spectra of the synthesized geopolymers doped with Nd^3+^ show interesting optical properties at 900 °C; the geopolymer matrix completely disintegrates and an amorphous phase with a rare-earth precipitate appears.

## 1. Introduction

In order to solve the problem of the greenhouse effect and reduce the excessive presence of carbon dioxide (CO_2_) in the atmosphere, it is imperative to allocate funds for the acquisition of environmentally sustainable materials and cutting-edge technologies that leave a significantly smaller carbon footprint [1]. In the 1970s, Joseph Davidovits achieved a remarkable milestone by discovering a highly sustainable cementitious substance that could potentially replace the widely used Portland cement (OPC) without any adverse impact on the environment. Geopolymers have been observed to possess a range of advantageous characteristics compared to traditional Portland cement. These include rapid solidification, enhanced mechanical strength, high resistance to extreme temperatures, and other beneficial practical properties [2,3]. This breakthrough was a significant advance in the field of environmentally friendly building materials [4]. After the inorganic polycondensation reaction is completed, a highly sustainable cementitious material known as geopolymer is produced. This substance is recognized for its eco-friendliness and is a notable alternative to conventional cement materials. Natural clay, waste, and by-products are widely used as alternative aluminosilicate materials for the synthesis of geopolymers; the use of these products, in addition to obtaining environmentally friendly material, also reduces construction costs [5]. According to their composition, geopolymers are a class of amorphous materials consisting of cross-linked [AlO_4_]^−^ and [SiO_4_] tetrahedra units, and the negative charge aluminum in fourfold coordination is balanced by alkali metal or alkaline earth metal cation, such as Na^+^, K^+^, Mg^2+^_,_ and Ca^2+^ [6,7,8,9].

Similar to geopolymers, alkali metal hydroxides are involved in many processes during alkali-activated reactions, including accelerating the dissolution of aluminosilicates, stabilizing the solution species and colloids, reducing the electrostatic repulsion between the anions, and promoting gel formation and rearrangement [10]. Geopolymers form an aluminosilicate matrix into which metal ions or other toxic substances can be incorporated, and their structure resembles a closed cage that includes a combination of ring molecules. This structure can separate metal ions or other harmful substances and trap them inside the cage. Extensive research has yielded numerous findings regarding the immobilization of toxic heavy metals through incorporation into alkali-activated structures [11,12,13,14,15,16]. The use of rare-earth elements is increasing in the industry, and in recent decades they have become the “new pollutants” [17]. Due to their unique and interesting properties, rare-earth oxides are widely used in various research fields. As the extraction and processing of rare-earth minerals continue, their potential for environmental pollution is on the rise. Moreover, the utilization of phosphate fertilizers may exacerbate this issue, leading to an even higher concentration of rare-earth elements in soil, particularly in regions used for agricultural purposes [18,19].

Various studies have shown that around 85 tons of neodymium (Nd) were released into the environment in the Netherlands in 1994 during the production of phosphate fertilizers [20]. Similar amounts of rare earth can also be released into the environment by petroleum refining [21]. The selection of neodymium oxide (Nd_2_O_3_) for electronic applications was based on its high κ value, ability to provide good step coverage and strong dielectric strength [22]. An additional issue that arises when rare-earth elements are released into the environment is that plants can uptake them from the soil, as their ionic radius is similar to that of calcium [23].

To mitigate the negative consequences that may result from the excessive presence of rare-earth elements in the environment and the organisms that dwell within them, it is crucial to adopt methods that immobilize these elements within geopolymer structures. Despite the significant research conducted on geopolymers and the increasing attention being paidto their possible uses, there is still a pressing need to acquire a thorough comprehension of these materials and the complexities of the alkali activation process to maximize their benefits. In the research work of Miao Liu and others, it was shown that different types of waste have different effects on the microstructure and macro-properties of alkali-activated materials and that, by optimizing the fineness of the waste, a sustainable material with good mechanical hardness can be achieved [24].

The rapid growth of urban populations and construction has resulted in the generation of vast quantities of construction waste. However, the conventional approach to disposing of urban construction waste in landfills has led to various social and environmental problems [25]. Apart from industrial waste, another problem is construction waste, such as concrete and brick waste fines; waste could be reduced by recycling and repurposing waste materials, which could help to mitigate the environmental effects and preserve natural resources [25]. Approaches such as nanotechnology have a significant potential to advance the construction industry, particularly in concrete production [26]. By applying nanotechnology to concrete production, researchers can better understand the material’s behavior, leading to improved mechanical properties and strengthening the concrete’s structural framework [26].

The main emphasis of this paper is a detailed examination of the thermal treatment process of geopolymers doped with rare-earth elements, specifically Nd_2_O_3_, and the subsequent analysis of the structural and chemical properties of the resulting geopolymer samples.

## 2. Materials and Methods

Geopolymers are synthesized in the reaction of metakaolin with an alkaline solution of sodium silicate. The activating solutions were prepared by dissolving 12 M sodium hydroxide powder and sodium silicate in an appropriate ratio (volume ratio Na_2_SiO_3_/NaOH = 1.5). Commercially available Nd_2_O_3_ powder was mixed during the process of alkali activation in geopolymer paste. The two weight fractions of oxide (1% and 5%) were used in the preparation of geopolymer samples. Metakaolin/Nd_2_O_3_ were mixed and left at room temperature for one day. After that, the mixture was kept at 60 °C for an additional two days in covered molds and subsequently aged at room temperature in controlled conditions for 28 days.

### 2.1. Diffuse Reflectance Infra-Red Fourier Transform Spectroscopy (DRIFT)

Diffuse reflectance infra-red Fourier transform spectroscopy (DRIFTS) is a cheap, fast and non-destructive way of evaluating clay minerals and their products. Drift spectra were obtained using the Perkin-Elmer FTIR spectrometer. Approximately 5% samples were dispersed in oven-dried spectroscopic grade KBr with the refractive index of 1.559 and particle size of 5–20 µm. Background KBr spectra were obtained, and spectra were rationed to the background. The spectra were scanned at 4 cm^−1^ resolution and collected in the mid-IR region from 4000 to 400 cm^−1^.

### 2.2. Transmission Electron Microscopy (TEM)

Characterization and investigation of the micro-nano-samples’ structure was carried out by TEM, using an FEI Talos F200X microscope operating at 200 keV. An CCD camera with a resolution of 4096 × 4096 pixels was used for acquiring micrographs using the User Interface software package. The geopolymer samples were also further analyzed using scanning transmission (STEM) mode with energy-dispersive spectrometry (EDS). The EDS detection system was used to determine the presence of doping species of the Nd in geopolymer matrix. High-angle annular dark-field (HAADF) imaging was used in nanoprobe-TEM mode with a camera length of ~200 nm, using the standard annular dark-field detector. The powder samples were prepared by standard rinsing and diluting in ethanol to a sufficient concentration to trap the geopolymer powder on the TEM grid, dried in air, and then transferred to microscope.

### 2.3. Scanning Electron Microscopy (SEM)

Field emission scanning electron microscope (FESEM, FEI Scios 2, Dual Beam system) was employed for morphological examinations. The samples were attached to a sample holder using double-sided copper tape. The micrographs were taken at an acceleration voltage of 15 kV and a chamber pressure of approximately 9 × 10^−5^ Pa.

### 2.4. UV/VIS

UV-VIS measurements were performed on a SHIMADZU UV-2600i spectrophotometer. The reflectance of the sample was measured in a wavelengths range from 250 nm to 1000 nm with an interval of 0.5. The software used for this measurement and data processing was LabSolutions UV-Vis, ver. 1.12.

### 2.5. X-ray Photoelectron Spectroscopy (XPS)

XPS analysis was performed using a SPECS instrument for detailed chemical composition characterization using X-ray induced photoelectron spectroscopy. Photoelectron emission was excited by monochromatic Al Kα line with a photon energy of 1486.67 eV. Detailed spectra of the main photoelectron lines were taken in the fixed analyzer transmission mode with a pass energy of 20 eV (FAT 20), an energy step of 0.1 eV and a dwell time of 2 s. Charging compensation was performed using an electron flood gun and the constant current and voltage. The binding energy axis was adjusted according to the position of the carbon C1s line. The survey spectra were performed according to the characteristic spectral line intensities. Specific atomic sensitivity factors for each analyzed element were used to eliminate the background lines provided by the manufacturer. The photoelectron lines were fitted to peaks using appropriate software package.

## 3. Results and Discussion

### 3.1. DRIFT Analysis

**Figure 1:**
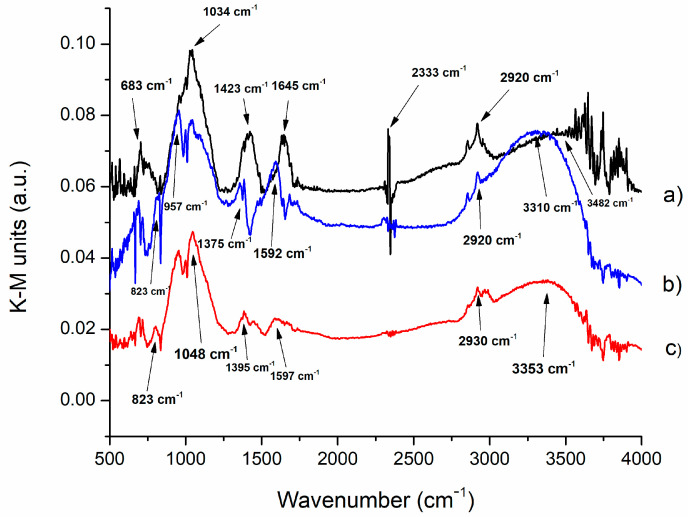
DRIFT analysis of GT: (**a**) GT_1_ treated at 300 °C, (**b**) GT_3_ treated at 600 °C and (**c**) GT_5_ treated at 900 °C.

**Figure 2:**
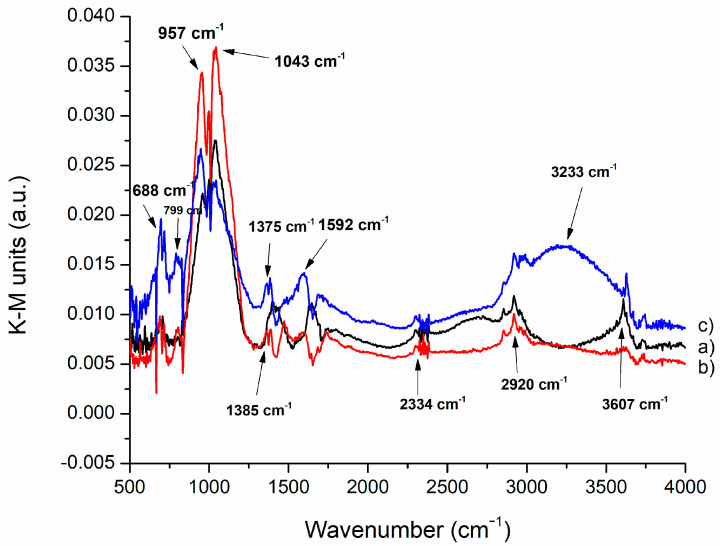
DRIFT analysis of GT: (**a**) GT_2_ treated at 300 °C, (**b**) GT_4_ treated at 600 °C and (**c**) GT_6_ treated at 900 °C.

The method employed to establish the structural characterization of alkali-activated materials utilizated Diffuse Reflectance Infrared Fourier Transform (DRIFT).According to Smith’s “Fundamentals of Fourier Transform Spectroscopy”, the graphical representation of Kubelka–Munk (KM) is significant for performing a quantitative analysis of DRIFT spectra. The absorption values (in arbitrary units) were derived from the diffuse reflectance measurements by applying the Kubelka–Munk function (f (R) = (1 − R)^2^/2R). Data manipulation was carried out using Microsoft Excel. The results of all samples are shown in Figure 1 and Figure 2. Figure 1 shows the results of thermally treated samples with the addition of 1% Nd, while Figure 2 shows the results of thermally treated samples with the addition of 5% Nd.

Figure 1 shows thermally treated geopolymers and their DRIFT spectra. As seen, the vibrational bands are very similar, indicating that there have been no significant changes in the chemical structure of the geopolymers. However, a more detailed analysis of individual bands reveals some interesting changes. The bands at 3482 cm^−1^, 3310 cm^−1^ and 3353 cm^−1^ are present in all geopolymers and originate from O-H stretching vibrations. This band is due to the O-H vibrations in silanoil (Si-OH) and bridging (Si-OH-Al). The presence of a band at 2920 cm^−1^ and 2930 cm^−1^ indicates the possibility of the presence of residual organic matter of aliphatic structure. Confirmation of O-H stretching vibrations is provided by a band at 2333 cm^−1^. Bands at 1592, 1597, and 1645 cm^−1^ are attributed to O-H bending vibrations. The bands at 1395, 1375 and 1423 cm^−1^ belong to the symmetric vibration of O-C-O from CO_3_^2−^ [27]. Increasing the temperature during thermal treatment leads to a reduction in the intensity of the vibrational band, indicating some changes in this part of the chemical structure of the geopolymers.

During alkali activation, a tetrahedral coordination of Si-O-T (T = Al or Si) is formed, which is confirmed by the bands at 1034 and 1048 cm^−1^ [28,29]. This band also changes in intensity with increasing temperature, indicating that this structure also changes during thermal treatment. The Si-O bending vibration occurs at the vibrational band of 957 cm^−1^ [30]. The band at 823 cm^−1^ belongs to the symmetric vibrations of Nd-O-Nd, while the band at 683 cm^−1^ belongs to Nd-O-Si vibrations [31]. These bands also show some changes in intensity with increasing temperature, which may be due to changes in the chemical structure of the geopolymers.

On the second graph shown in Figure 2, thermally treated geopolymers containing 5% Nd_2_O_3_ are presented, showing a similar spectrum pattern as in Figure 1. Bands at 3233 and 3607 cm^−1^ are attributed to O-H stretching vibrations. The bands at 1592 cm^−1^ and 1375 and 1385 cm^−1^ were observed, which can be attributed to Si-O and O-C-O bonds, respectively. These bands were also visible in samples containing 1% Nd_2_O_3_ but at slightly higher values. A band at 1043 cm^−1^ was also observed in Figure 2, corresponding to the Si-O-T (T = Al or Si) vibrations in tetrahedral coordination. Si-O bending vibrations occur at the vibrational band of 957 cm^−1^. Symmetric vibrations of Nd-O-Nd are found at 799 cm^−1^. The band at 688 cm^−1^ was observed, corresponding to the Nd-O-Si bond, which had a higher value compared to the values in the first graph.

### 3.2. TEM Analysis

**Figure 3:**
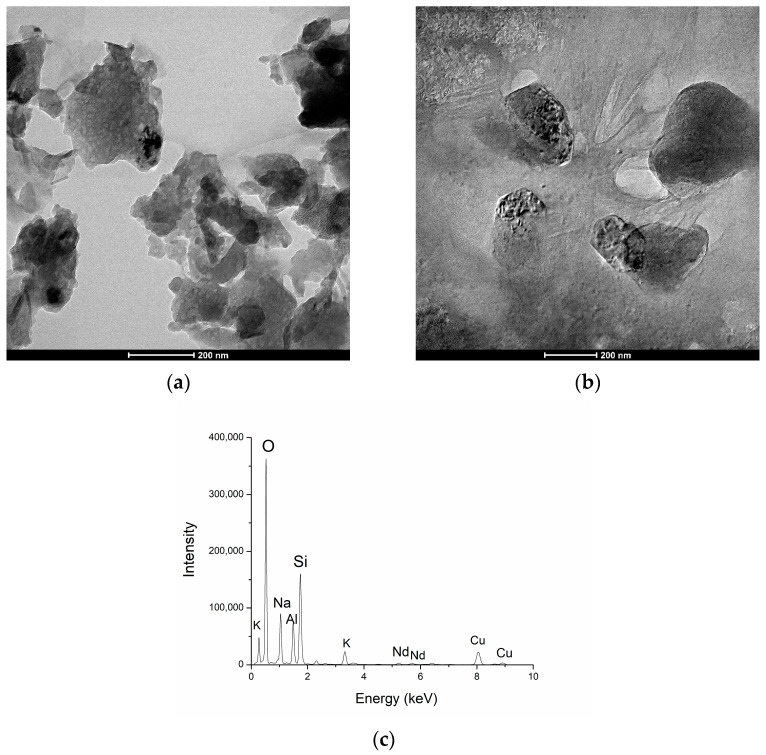
TEM analysis of GP: (**a**) with 1% of Nd, (**b**) with 5% of Nd and (**c**) corresponding EDS spectra of GP with 5% of Nd.

The geopolymer samples doped with 1% and 5% of Nd_2_O_3_ are characterized using transmission electron microscopy (TEM). The magnification used for both samples was 55 kx, Figure 3a,b, and corresponding X-ray energy dispersive spectrum (EDS) are shown in Figure 3.

Figure 3a exhibited a unique structure characterized by spherical formations dispersed throughout a sponge-like microstructure containing nano-sized pores [32]. In Figure 3a, the existence of two different phases is clearly visible, which are clearly different in color, forming light and dark fields. Lighter-colored grains present Al_2_O_3_. The dark colored grains mostly present SiO_2_ as quartz. In Figure 3a, a very uniform distribution of bright grains in larger crystallites can be observed. This indicates the homogeneity of the geopolymeric matrix, where Al_2_O_3_ is dispersed in the form of nanoparticles with a diameter of 5–10 nm in larger crystallites that form together with the quartz phase SiO_2_. The distribution of the quartz phase is not homogeneous, but there are areas with a lower and higher concentration of quartz.

Larger grains are in the range 30–80 nm and the occurrence of aggregation is noticed, which leads to the formation of larger grains of about 300 nm. Figure 3b shows a geopolymer doped with 5% Nd. The structural differences are obvious compared to the geopolymer doped with 1% Nd. In this case, large grains appear that have coalesced due to the diffusion processes. Nd is found, together with the Al_2_O_3_ and SiO_2_ phases, in large crystallites; the alumino-silicate phases are located in the middle of the grain, while Nd tends to separate and diffuse towards the grain boundaries due to obvious thermodynamic stability. The presence of 5% Nd as a dopant is confirmed using EDS analysis, as in Figure 3c, where the alpha and beta lines of Nd are visible as small peaks in the middle of the energy scale, while Si and Al peaks dominate the spectrum.

### 3.3. SEM Analysis

**Figure 4:**
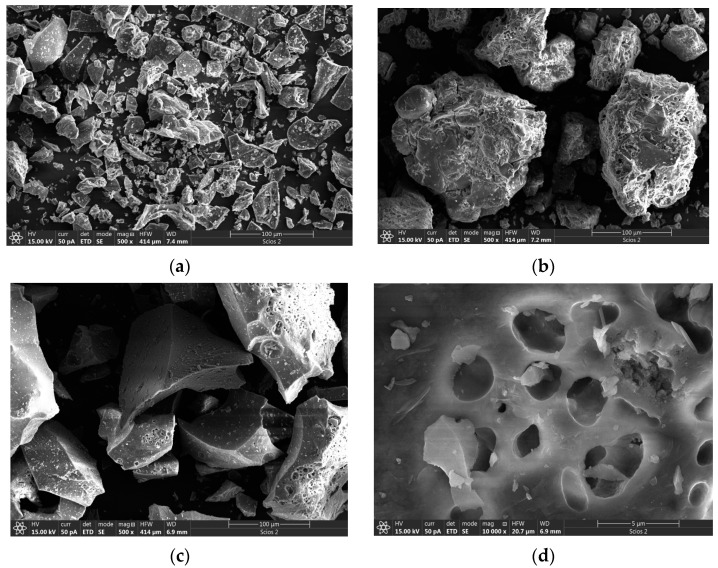
SEM analysis of aluminosilicate matrix with 1% Nd (**a**) thermally treated at 300 °C, (**b**) thermally treated at 600 °C (**c**,**d**) thermally treated at 900 °C.

The thermal treatment of geopolymers doped with 1% Nd is presented in Figure 4. The samples were treated at 300 °C, 600 °C, and 900 °C, respectively. At the lowest thermal treatment temperature of 300 °C (Figure 4a), the geopolymer increases the stiffness while extracting crystalline moisture. The geopolymer shows clear crystal boundaries, as well as very well-defined grain boundaries, in polycrystalline grains. The geopolimer doped with 1% Nd shows a stable state without additional effects on both the surface and the bulk of polycrystalline grains. With an increase in thermal treatment temperature to 600 °C, (Figure 4b) the propagation of pores occurs. This means that the overall temperature of geopolymer has increased enough to expell all crystalline water and continues to act by opening small pores and cracks in polycrystalline grains. The appearance of small pores is the consequence of the pile-up of the material due to the increased temperature. In addition to the release of crystalline water, there is an interruption of some alumosilicate bonds that further enhance the creation of micropores.

At a temperature of 900 °C, the propagation of pores increases. Figure 4c shows the spread of pores to the whole grain of the geopolymeric base, with a clear tendency toward further advancement. Figure 4d clearly reveals the appearance of larger pores in the geopolymer matrix. The high temperature led to an interruption of Al-SiO bonds, and thus to the creation of pore dimensions of about 2 μm in diameter, which results in an increased geopolymer porosity with increasing temperature. The distribution of pore size is almost uniform. Compared to the work of Aref A. Abadella et al., a trend of increasing porosity with an increasing percentage of dehydrated cement powder is observed, while in this work, porosity increases with temperature changes [33]. The porosity of the material can also be increased by the addition of date palm fibers, as shown in the work of Yasser E. Ibrahim et al. [34].

**Figure 5:**
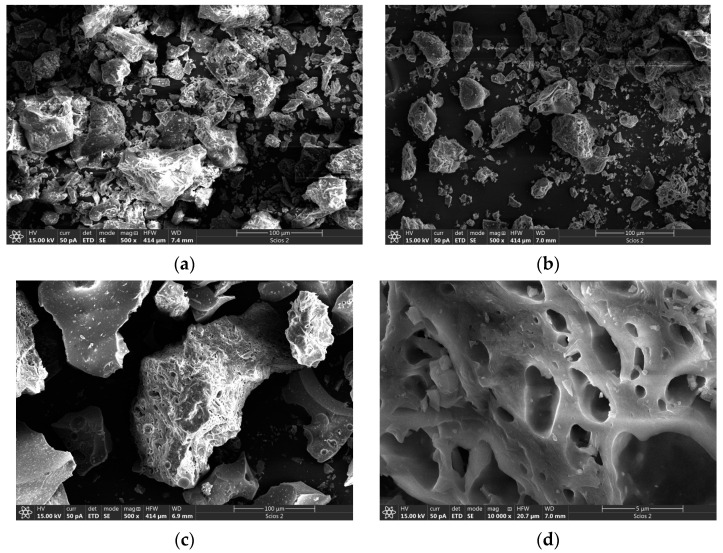
SEM analysis of aluminosilicate matrix with 5% Nd (**a**) thermally treated at 300 °C, (**b**) thermally treated at 600 °C, (**c**,**d**) thermally treated at 900 °C.

Geopolymer samples doped with 5% Nd and thermally treated at 300 °C, 600 °C and 900 °C are shown in Figure 5. At a temperature of 300 °C, the effect of grain compact and expulsion crystalline moisture is obvious, as shown in Figure 5a. Compact and consistent grains, as well as stable crystals, are formed at a temperature of 300 °C. At a temperature of 600 °C, the propagation of pores occurs. The pores are unevenly distributed and appear on certain parts of the crystal grains. We can assume that there is a more intense pore occurrence in places enriched by neodymium; Figure 5b–d reveals the full size and the pore distributions in the geopolymer matrix. At 900 °C, an intense interruption to Al-SiO bonds occurs, and materials are collected in places that are enriched by neodymium. The delocalization of pores in places with an incorporated neodymium, as well as their inequalities in size, are a consequence of the geopolymer incorporation with 5% Nd. The pores have an irregular elliptical shape of diameter up to 5 μm with a tendency toward further spread. The increased concentration of neodymium conditioned the inhomogeneous distribution of pores, as well as their shape and size.

### 3.4. UV/VIS Analysis

**Figure 6:**
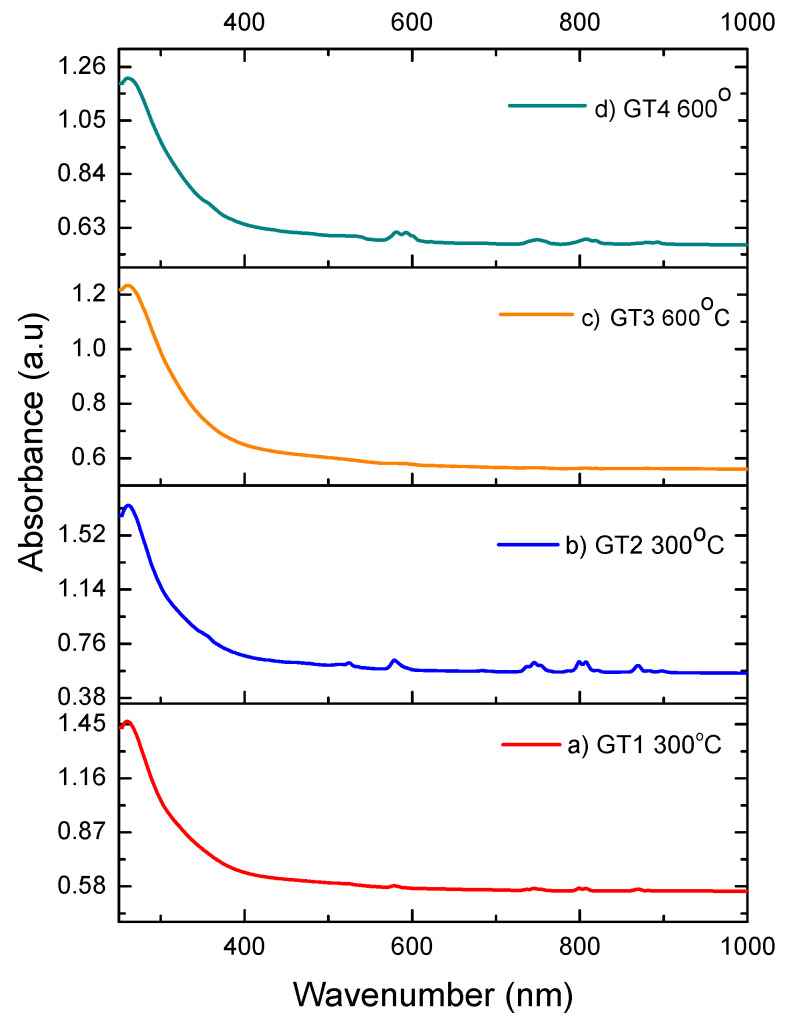
UV/Vis spectrum of thermally treated GP: (**a**) 1% Nd 300 °C, (**b**) 5% Nd 300 °C, (**c**) 1% Nd 600 °C and (**d**) 5% Nd 600 °C.

Figure 6 shows the UV/Vis spectra of thermally treated geopolymers at 300 °C and 600 °C, doped with 1% Nd and 5% Nd. All samples were recorded in the range from 200 nm to 1000 nm.

In the first graphic in Figure 6a, the absorbance is 1.45 at a wavelength of 260 nm; this absorbance can be attributed to the structure of the synthesized geopolymers. Using TEM analysis, a homogeneous distribution of Al_2_O_3_ is observed; we assume that this absorbance can be attributed to the photoexcitation of electrons from the valence band and conduction band [35]. Very small peaks at 580 nm, 745 nm, 803 nm, and 871 nm are also noticeable, which are attributed to the Nd^3+^ with which geopolymer samples were doped [36]. These peaks are lost in the sample that was thermally treated at 600 °C, as shown in Figure 6c; the assumption is that diffusion during the thermal treatment at temperatures above 300 °C changes the thermodynamics of Nd^3+^.With an increase in temperature to 600 °C during the thermal treatment, the absorbance decreases to 1.25 because the structure of the geopolymer changes; this is visible on the SEM, where we can see that the pores expand and crachs occur in the polycrystalline grains.

The spectra of samples doped with 5% Nd are shown in Figure 6b,d. Compared to samples doped with 1% Nd, a large difference can be observed in the appearance of the UV/Vis spectrum. The absorbance of the sample treated at 300 °C is 1.7, which can be attributed to Al_2_O_3_ and the effect of Nd on the geopolymer structure. Bands appear at 528 nm, 579 nm, 745 nm, 803 nm, and 869 nm; the absorbance is much lower and is attributed to the Nd^3+^ ion doped in the thermally treated geopolymer. Both samples doped with 5% Nd, as well as samples doped with 1%, which were thermally treated at 600 °C, show a drop in absorbance to 1.2 at 260 nm. In contrast to the samples with 1% Nd, in samples with 5% Nd, there was no complete disappearance of the bands at slightly higher wavelengths; therefore, bands at 589 nm, 749 nm, 809 nm, and 891 nm are visible, which is also attributed to the Nd^3+^ ion.

### 3.5. XPS Analysis

**Figure 7:**
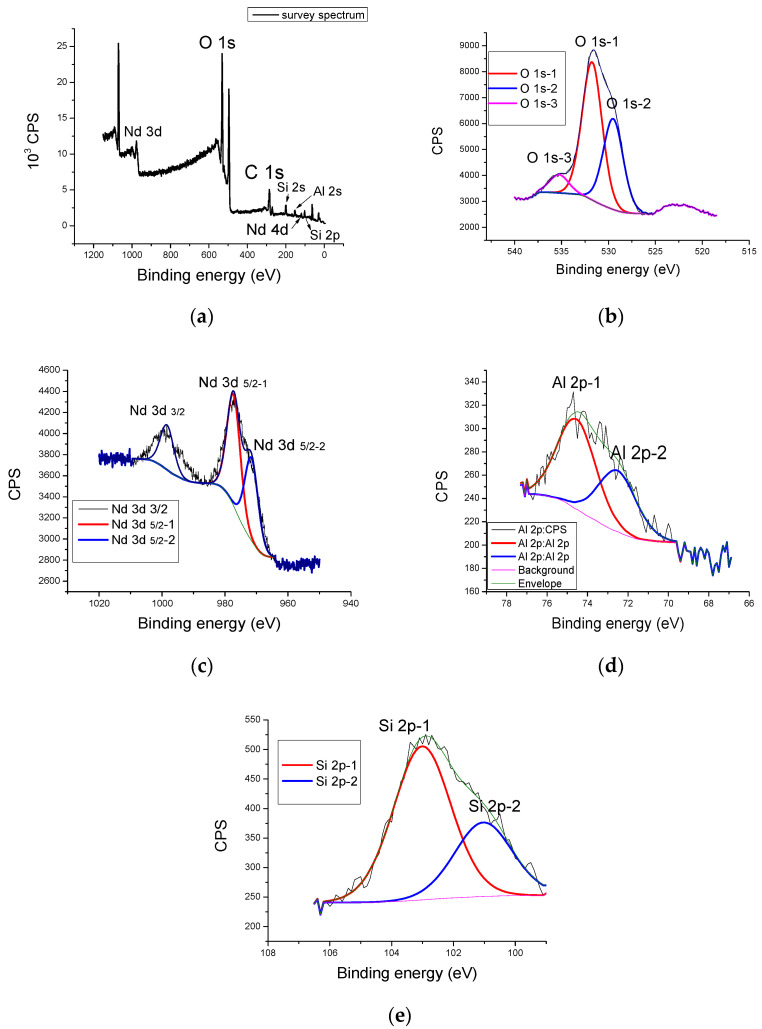
XPS spectrum of GP with 5% Nd: (**a**) survey spectrum, (**b**) O 1s, (**c**) Nd 3d, (**d**) Al 2p and (**e**) Si 2p.

Figure 7a presents a survey spectrum of a geopolymer doped with 5% neodymium, where a Nd 4d spectral line appears. The most dominant peaks in Al 2p and Si 2p spectral lines is observed in the low-energy part, together with O1s and C1s. In the high-energy part of the spectrum (around 1000 eV), a complex peak in the neodymium Nd 3d spectral line is visible.

The means by which oxygen binds in the GP matrix is shown in Figure 7b using the detailed spectrum of the O 1s deconvoluted spectral line. The deconvolution of the oxygen spectral line makes three contributions. The first, most pronounced (O 1s-1) can be found at 531.2 eV of the binding energy and represents the main phase of Al_2_O_3_. The second contribution, with a lower intensity (O 1s-2),can be found at 529.3 eV. This can be attributed to a SiO_2_ and quartz-related compounds. The third and the least pronounced peak (O 1s-3) at 535.4 eV of binding energy belongs to formation of complex aluminosilicate compounds, created as a byproduct of alkaline activation (CaAl_2_O_4_ and SiAl_2_O_4_). The most pronounced oxygen peak appears at 531.8 eV O 1s-1;this can be attributed to Al_2_O_3_. The second contribution of O 1s-2 to 529.3 eV is mainly attributed to SiO_2_. In Figure 7b, the between the Al_2_O_3_ and SiO_2_ phases can be determined. This is 2:1, which agrees with the previously known data.

Neodymium (III) oxide was used as a doping source, leading to the formation of three dominant contributions of the neodymium spectral lines shown in Figure 7c. In the geopolymer doped with 5% neodymium, one can notice the Nd 4d spectral line, together with the most pronounced Nd 3d spectral line, clearly visible on the survey spectrum, as shown in Figure 7a. The presence of the Nd 4d spectral line in the low-energy scale of the spectrum is a consequence of the amount of Nd concentration. Previous investigations showed that, at a lower dopant concentration, the Nd 4d line does not appear [37]. Detailed spectra od neodymium 3d and 4d spectral lines reveal the more pronounced contribution of Nd 3d 5/2-1 to 998.9 eV belonging to Nd (OH)_3_, as well as its contribution to 977.9 eV (Nd 3d 5/2-2), and can be attributed to pure Nd_2_O_3_, as shown in Figure 7c. The ratio of the amounts of hydroxide and oxide phase offers information about the reaction rate of neodymium in the geopolymer matrix during alkaline activation.

The detailed spectra of aluminum 2p shown in Figure 7d reveal the existence of Al_2_O_3_ at the energy rate of 73.9 eV—Al 2p-1. This more pronounced peak is attributed to amorphous Al_2_O_3_ and less pronounced crystalline Al_2_O_3_, and can be seen at a binding energy of 71.4 eV (Al 2p-2). This can be attributed to the alpha phase crystal structure. The summary ratio between amorphous and crystalline Al_2_O_3_ is 4: 1, which is a consequence of the alkaline activation process where, due to the reaction conditions and the concentration of the alkaline activator, a preferential formation of the amorphous phase occurs.

A detailed spectral analysis of the Si 2p line is shown in Figure 7e. This clearly shows a dominant peak at 102.9 eV (Si 2p-1), attributed to the SiO_2_ and Al_2_OSiO_4_ phases. A less pronounced contribution peak is located on 101.5 eV (Si 2p-2), which can be attributed to the molecular sieve (zeolite 3A) of NaAl_2_SiO_4_ [38]. This is proof that it is possible to obtain zeolite structures in amorphous form by the alkaline activation of geopolymers with the presence of rare-earth ions.

## 4. Conclusions

- Thermal treatment and Nd_2_O_3_ doping significantly impacts the chemical structure and physical properties of geopolymers, as observed through DRIFT spectra, TEM and SEM analysis.
- XPS analysis reveals the successful doping of Nd in geopolymers and provides information on the bonding and formation of Al_2_O_3_ and complex aluminosilitace compounds.
- Nd^3+^ doping in geopolymers has promising potential for modifying their optical properties for use in optoelectronics and photonics applications, although further studies are needed to optimize synthesis conditions.
- Increasing temperature during the thermal treatment of geopolymers doped with Nd results in changes to their physical properties, including an increase in stiffness and propagation of pores, leading to cracks in polycrystalline grains.
- The alkaline activation of geopolymers in the presence of rare-earth ions can lead to the formation of amorphous zeolite structures, highlighting the potential for their use in various industries.
- Overall, these findings contribute to the understanding of the influence of thermal treatment and doping on geopolymers, with implications for the development of more efficient materials for construction and environmental remediation.

## Data Availability

Not applicable.
